# Clinical Gleanings

**Published:** 1895-06

**Authors:** 


### CLINICAL GLEANINGS.

The treatment recently advocated by Dr. Lusson for "Azoturia," and published in the February number of this JOURNAL, has been successfully used in several cases by one of the readers of this JOURNAL. We should be glad to publish the experience of others.

Dr. James A. Waugh has observed that cases of tetanus which are slow in development recover under approved treatment in a much larger per cent. than those which arise rapidly. This would well apply to two cases recently treated by one of the editors of this JOURNAL, in which recovery was obtained. Let us hear from others ; it is of much importance.

Dr. W. Horace Hoskins reports the case of a large brown gelding, used in a coal wagon, prostrated on the street with heat-stroke ; temperature 106° when reached, with violent movements ; after the free use of water from a hose, and the administration of diffusible stimulants, was subsequently removed to his sanitarium with ambulance, and same treatment continued until the temperature reached the normal point. Bran mashes only were given as food, with grass and small quantity of hay. During the succeeding seventy-two hours forty large intestinal round-worms were voided, all dead. Was it not the high temperature that proved the effective vermifuge?

